# The Association of Food Consumption Scores, Body Shape Index, and Hypertension in a Seven-Year Follow-Up among Indonesian Adults: A Longitudinal Study

**DOI:** 10.3390/ijerph15010175

**Published:** 2018-01-22

**Authors:** Emyr Reisha Isaura, Yang-Ching Chen, Shwu-Huey Yang

**Affiliations:** 1 School of Nutrition and Health Sciences, College of Nutrition, Taipei Medical University, Taipei 11041, Taiwan; d507103003@tmu.edu.tw (E.R.I.); melisa26@gmail.com (Y.-C.C.); 2 Department of Family Medicine, Taipei City Hospital, ZhongXing Branch, Taipei 10341, Taiwan; 3 Nutrition Research Center, Taipei Medical University Hospital, Taipei 11041, Taiwan

**Keywords:** food consumption score, a body shape index, hypertension, mediation analysis

## Abstract

*Aims*: The concept of food security and its association with chronic diseases are both well-established. During the years within the scope of the study, there was a significant increase in the body shape index (ABSI) of Indonesian adults. This study tested the hypothesis that the association between food security and chronic diseases is mediated, in part, by ABSI. *Methods*: Data was obtained from 2156 Indonesian adults using the Indonesia Family Life Survey (IFLS) in 2007 and 2014. Longitudinal study participants were interviewed face-to-face for dietary intake data using the food frequency questionnaire (FFQ). Food security, a concept developed by the World Food Programme (WFP), was calculated based on a food consumption score analysis using the FFQ. A generalized estimating equation (GEE) and a Sobel–Goodman test were used to test the hypothesis in this study. *Results*: The food consumption score was negatively associated with ABSI. It was also negatively associated with systolic blood pressure (*p* < 0.001). In a formal mediation analysis, ABSI significantly mediated the pathway between the food consumption score and systolic blood pressure (*p* < 0.001). *Conclusions*: The effect of food security on hypertension is mediated through body shape. Strategies to improve the prevention of hypertension among adults may need to take the ABSI and food security, along with nutrition education, into account.

## 1. Introduction

Food insecurity is associated with obesity. Food insecurity means the inability of individuals to consume a better quality or more diverse diet [1,2,3]. Measurements for food security are numerous but the concept itself is rather elusive [4]. To measure food security, the United Nations World Food Programme (WFP) focuses on diet diversity and food frequency. In general, a better quality and more diverse diet is characterized by higher nutrient density; this describes a food group’s quality in terms of (1) caloric density, (2) macronutrient and micronutrient content, and (3) actual quantities eaten [4]. Furthermore, food insecurity is related to chronic diseases such as hypertension and cardiovascular disease [5], indicating that the inability of a person to have a balanced meal is an obstacle to living a healthy life. 

**Figure 1:**
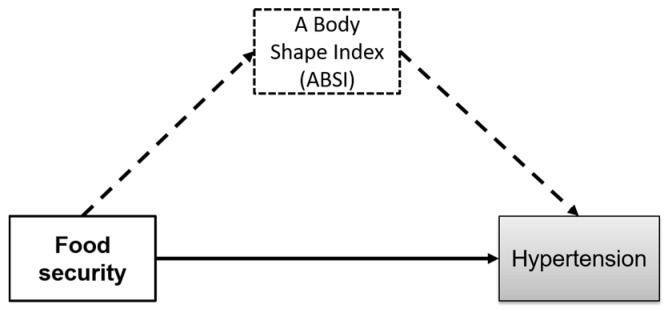
A Body Shape Index may mediate the relation between food security and hypertension.

Hypertension (HTN) has been a global burden disease since the 1990s; as such, a systolic blood pressure (SBP) level is an important measurement in guiding prevention or intervention policies [6]. Hypertension is one of ten leading causes of death in lower-middle income countries such as Indonesia. In 2016, Helble and Aizawa reported that about 45% of all respondents over the age of 39 suffered from hypertension [7]. An SBP level of at least 110 mmHg is associated with many cardiovascular diseases (CVD) [8], and global obesity may exacerbate SBP in some populations [9,10]. In the results from the Global Burden of Disease, Injuries, and Risk Factor study of 2015 (GBD 2015), Wang and colleagues highlighted a stronger association between SBP and global disease burden compared to the association between global disease burden and either obesity or smoking habit [11]. There have been few studies evaluating the association between food security and hypertension [5,12]. This study is the first to explore the pathway from food security to hypertension and the mediating effect of a body shape index (Figure 1). 

## 2. Materials and Methods

### 2.1. Study Design and Participants

Data was obtained from the Indonesia Family Life Survey (IFLS), an ongoing longitudinal study initiated in 1993 [13]. The population of this study consisted of women and men aged 18–65 years who participated in the 2007 and 2014 follow-up. In total there were 22,459 participants; 59.1% of the adult participants who participated in 2007 (12,335) completed the food frequency questionnaire (FFQ) record. In 2014, 24,744 (57.5% of the adult participants) participated in the study, and 8311 completed all the IFLS4 and the IFLS5 FFQ records (33.6%). Furthermore, participants who had two FFQ records; no missing data on the anthropometric measurement; did not use hypertension lowering medication; and had not been diagnosed as having cancer, cardiovascular disease, or diabetes before 2013 were included in this study. Therefore, we used dietary data from 2156 individuals, 25.9% of total IFLS5 participants. These participants had completed the sociodemographic and anthropometric measurements data (i.e., height, weight, waist, and hip circumference). The IFLS went through an institutional review board (IRB) review and was approved by RAND corporation, as well as the institutions in Indonesia that sufficiently and properly reviewed the study with respect to human subject issues [13,14].

### 2.2. Food Security Assessment

In practice, food security correlates with food frequency and dietary diversity proxy indicators with regards to the access of a sufficient quantity of food [15,16,17]. Food security, as developed by the WFP, covered food frequency and dietary diversity in the food consumption analysis. Food consumption analysis entails using a food frequency questionnaire (FFQ) and food group weighted score to determine a resulting food consumption score (FCS) [4,18]. Therefore, we used the FCS to assess food security. The FFQ was conducted in face-to face interviews to collect dietary data; 10 food items (green leafy vegetables, carrot, banana, papaya, mango, sweet potato, meat, fish, eggs, and dairy) were reported in IFLS4 and IFLS5. The ten food items were then grouped into five food groups: fruits, vegetables, staples, dairy, and proteins. The fruit group was comprised of banana, papaya, and mango; the vegetables were comprised of green leafy vegetables and carrots; the staple group was comprised of sweet potato; dairy was defined as the dairy product consumed by the participants; and proteins were defined as any type of meat, fish, and eggs. The FCS principal guideline for determining food group weights is to use the nutrient density of the food groups. The highest weight was attached to foods with relatively high energy, good quality protein, and a wide range of micronutrients that can be easily absorbed [4,18]. The FCS continuous data obtained from the food consumption analysis was categorized into three food consumption groups (FCG) to determine the food security (FS) level. Food consumption groups are categorized into (1) “poor” if FCS falls below 21, (2) “borderline” if FCS ranges from 21 to 35, and (3) “acceptable” if FCS is above 35. Based on the FCG, the FS level was divided into two groups: (1) “food insecure” if the FCG is categorized in the borderline or poor groups and (2) “food secure” if the FCG is categorized in the acceptable group [18]. The continuous food consumption scores were used in the descriptive and correlation analyses. To analyze upward and downward proportions of FCS change from 2007 to 2014, we categorized the FCS changes into three subcategories: (1) “increased” if the FCS in 2007 is less than in 2014, (2) “decreased” if the FCS in 2007 is greater than in 2014, and (3) “maintained” if the FCS in 2007 is equal to that of 2014. The same categories were also used in the comparison of participant body shape index from 2007 to 2014.

### 2.3. Anthropometric Measurements

Trained nurses measured body weight, height, waist, and hip circumference during the data collection process. Anthropometric data was used in continuous and categorical forms. A high waist circumference (WC) was defined at >80 cm for women or >90 cm for men. Body mass index (BMI) was calculated as weight (in kilograms) divided by height (in meters) squared. BMI was categorized into nonoverweight if BMI < 25.1 kg/m^2^ and overweight if BMI > 25.1 kg/m^2^ [19]. A body shape index (ABSI) was calculated using formula below [20,21,22] in m^11/6^ kg^−2/3^.

$$\mathrm{ABSI}=\;\frac{\mathrm{WC}}{{\mathrm{BMI}}^{2/3}{\mathrm{Height}}^{1/2}}$$

For the purposes of this paper, we used ABSI as continuous data and classified it into four quartiles, with quartile 1 being the lowest and quartile 4 being the highest. A high ABSI indicates that compared to individuals of the same age and sex, a person’s WC is higher than average given their height and weight; this corresponds to a higher concentration of body volume centrally [22]. 

### 2.4. Health Outcome

Health outcome in this study is defined as whether the person has HTN. A participant is said to have hypertension if they have an SBP of ≥140 mmHg, a diastolic blood pressure (DBP) of ≥90 mmHg, and/or have been diagnosed with hypertension by health practitioners [23]. As categorical data, health outcome is divided into two categories: hypertensive and nonhypertensive.

### 2.5. Statistical Analysis

Participant characteristics for 2007 and 2014 are shown as mean and standard deviation (SD) for normally distributed variables; median (25th, 75th percentile) was used for FCS owing to its right-skewed distributions. The proportion of participants in the increase, maintain, and decrease FCG groups (meeting the three categories described earlier in the food security assessment) are displayed in proportions within the male, female, and total sample populations. A *t*-test was used to compare ABSI values among those who increased, decreased, or maintained their food consumption between 2007 and 2014. We used a generalized linear model to observe the association between independent variables, ABSI, and the health outcomes; this was calculated with the generalized estimating equation (GEE) test. In the GEE and mediation analysis tests, we combined data of the independent and dependent variables in 2007 and 2014. 

The role of ABSI in mediating the relationship between independent variables (i.e., FCS, FCG, FS) and health outcomes (i.e., SBP, DBP, HTN) was assessed using the Baron and Kenny three-step framework for formal mediation analysis. Aggarwal and colleagues have used the same steps in their former study [24]. The three steps are (1) the assessment of the independent variable and mediator, (2) the assessment of the mediator and health outcome, and (3) the assessment of the independent variable and health outcome. To formally assess step 3, both the first and the second association should be significant. If the results of steps 1, 2 and 3 are significant and the strength of the association between the independent and health outcome is reduced when the mediator variable is added to the model, there is a mediation between the independent variable and the health outcome [25]. This method is similar to analytical techniques frequently used by epidemiologists, and it tests the strength of the mediation using the Sobel–Goodman test [25]. The *p*-value of <0.05 was used to test for statistical significance. All analyses were conducted using STATA 12.1 (StataCorp LP, College Station, TX, USA).

## 3. Results

### 3.1. Participants Characteristics

Table 1 shows that the mean age of participants was 59(3) years for women and men. The mean ABSI was higher in women than men being 0.0843(0.0065) m^11/6^ kg^−2/3^ and 0.0817(0.0047) m^11/6^ kg^−2/3^, respectively. In 2007, men (83%) were more likely to have a smoking habit than women (6.94%). The prevalence of overweightness in women (37.42%) was higher than in men (16.91%). Abdominal obesity among women was also higher (56%) than among men (14.52%). The prevalence of hypertension in women (49.14%) was higher than in men (39.35%). In 2014, there was an increase in smoking habit prevalence among both women (from 6.94% to 9.11%) and men (from 83% to 85.77%). The prevalence of overweightness and of abdominal obesity in women was still higher compared to that in men. The prevalence of hypertension increased in both women (from 49.14% to 52.84%) and men (from 39.35% to 41.26%). Diabetes was found in 43 of the total participants, while coronary heart disease was found in 13 of the total participants, and stroke incidence was found in six participants by the end of follow-up study. The mean BMI, ABSI, and SBP for women was higher than those for men. The mean BMI, ABSI, and SBP significantly increased for both women and men (*p* = 0.016 to < 0.001). 

Table 2 shows the proportions of participants in each of the categories referring to the change in FCG. The percentage of participants in the poor FCG increased from 9.37% in 2007 to 23.84% in 2014. The percentage of participants in the borderline FCG was increased from 17.12% in 2007 to 32.10% in 2014. Participants in the acceptable FCG decreased from 73.52% in 2007 to 44.06% in 2014. There was significant difference in the proportion of women and men in the poor, borderline, and acceptable groups (*p* < 0.001). The mean of participants’ food consumption score per week decreased from 50.76 in 2007 to 33.77 in 2014. Overall, food consumption scores per week decreased 33.47% between 2007 and 2014 (*p* < 0.001). The mean ABSI based on the three FCG increased between 2007 and 2014. In Supplementary Table S1, there are 432 (20.04%) total participants who increased their FCS; on the contrary, 1685 (78.15%) of total participants decreased their total FCS between 2007 and 2014. There are 39 (1.75%) participants who maintained their FCS. There was no difference in the proportion of women and men in the increase, maintain, and decrease change groups (*p* = 0.329).

### 3.2. Correlation of Food Security, a Body Shape Index, and Hypertension

Table 3 shows the correlation of food security, ABSI, and hypertension. The GEE test between food security and hypertension shows a negative correlation between the independent variables (i.e., FCS, FCG, FS) and dependent variables (i.e., SBP, DBP and HTN) (*p* < 0.001). There is also a negative correlation between the independent variables and ABSI (*p* = 0.001–0.024). Moreover, there is a positive correlation between ABSI and the dependent variables (*p* < 0.001). 

### 3.3. Role of ABSI in Mediating the Relationship between Food Security and Hypertension

Table 4 shows mediation analysis with hypertension as the dependent variable, food security as the independent variable, and ABSI as the mediating variable. There is a negative correlation between food security and the body shape index (β = −4.94 × 10^−4^, 95% CI = −8.78 × 10^−4^, −1.09 × 10^−4^, *p* = 0.012). On the other hand, a negative correlation also found between food security and hypertension (β = −5.67, 95% CI = −7.09, −4.25, *p* < 0.001). A mediation analysis successfully tested a pathway between food security and hypertension. Based on the three-step framework of the mediation analysis, the study results show that the first step is successful; assessment of independent variables (i.e., FCS, FCG, and FS) and mediator variable (ABSI) are statistically significant (*p* = 0.024–0.001). The second step, an assessment of the independent variable (i.e., FCS, FCG, FS) and health outcomes (i.e., SBP, DBP, HTN) are statistically significant (*p* = 0.001 to < 0.001). The third step is also statistically significant (*p* = 0.002 to < 0.001), and the strength of the association between food security and hypertension was attenuated from −5.67 to −5.52 after a body shape index added to the model.

## 4. Discussion

The present study explored the relationships between food security and hypertension, as well as the mediation pathway by ABSI. As expected, the GEE analyses confirmed a positive association between ABSI and hypertension, whereas a negative association was found between food security and hypertension. Furthermore, mediation analyses confirmed that ABSI mediated a pathway between FCS and hypertension, as well as showed the direct and indirect influence between the variables. Several means of measuring food security have been applied in research and policy; this includes studies that assessed the mediators of food security based on health outcome. Former research studies have reported a cross-sectional association between food insecurity and hypertension, diabetes, heart diseases, or general health status [1,5,26,27,28,29]. Seligman and colleagues suggested food insecurity as a risk factor for hypertension and diabetes among 18–65 year-old adults [5]. This study’s results are in line with these findings; among Indonesian adults, food insecurity is associated with hypertension, as evident from the clinical evidence (i.e., SBP and DBP measurements). 

This study is the first to support a previous observation that a body shape index is an important index connected with food insecurity and hypertension. And we further discussed the mediating effect of body shape index. Wilde and Peterman [3] reported that food insecurity had a positive correlation with obesity, while there is a U-shaped curve for the association between food insecurity and weight change categories in women. Aggarwal and colleagues reported that people with low socioeconomic status tend to consume a high-energy density and poor nutritional diets with a low cost; this leads to an increase in energy intake and increased prevalence of overweightness [24,30,31,32,33,34]. The hypothesis that a high energy-dense and poor nutrient diet is associated with low FCS may be one of the mechanisms to explain the food security gap in relation to chronic disease [1,27,28,29,35,36,37]. This study uses a population-based sample which confirms these findings with repeated clinical evidence of hypertension (i.e., SBP and DBP); this also suggests that adults in the food insecure group (poor and borderline FCG) are more likely to have an increased mean BMI, waist circumference, and ABSI. This study had certain limitations. Firstly, the food consumption score used as an indicator of food security was based on the FFQs, which does not account for specific amounts of food intake [38]. However, using a food consumption score as a food security indicator has been done in several studies, and it captures both dietary diversity and food frequency [4,18,35,39]. Secondly, physical activity (PA) together with diet were both basic factors of body shape index, but we did not assess PA in our study period. However, prior studies reported that food insecurity is not associated with physical activity [40,41,42] and suggested that increased adiposity causes a reduction in physical activity, not vice versa [43,44]. Therefore, we did not consider adjusting physical activity. Thirdly, the study sample was representative of adult populations of the western part of Indonesia and not of the Indonesian population as a whole. However, the sample population characteristic represents a majority of the Indonesian demographic. 

Nonetheless, there are some implications for epidemiological studies and public health policy from the present findings. Firstly, food security is a critical, yet underappreciated, factor that may explain overweightness and hypertension amongst adults. The present study result can be attributed to the general consensus that the food consumption score is a direct proxy for hypertension prevention [28]. It becomes a supportive solution for a NCDs prevention. Additionally, for future studies aimed at improving diet diversity and food frequency towards the prevention of chronic diseases, ABSI can be used as an obesity indicator tool along with the body mass index. Secondly, improving dietary knowledge, within dietary diversity and quality constraints, should continue to be a focus of nutrition education programs and interventions to lower blood pressure measurements in the normal range. 

## 5. Conclusions

The effect of food security on hypertension is mediated through body shape. Strategies to improve the prevention of hypertension among adults may need to take the ABSI and food security, along with nutrition education, into account.

## Figures and Tables

**Table 1:** Characteristics of participants.

Variable	All (*n* = 2156)			Women (*n* = 1109)			Men (*n* = 1047)			*p* Value ^b^
	2007	2014	*p* Value ^a^	2007	2014	*p* Value ^a^	2007	2014	*p* Value ^a^	
Age (years), mean	52(3)	59(3)		52(3)	59(3)		52(3)	59(3)		
Education level, %			0.445			<0.001			<0.001	0.497
Low (<12 years)	1833(85.02)	1815(84.18)		992(89.45)	841(80.32)		989(89.18)	826(78.89)		
High (≥12 years)	323(14.98)	341(15.82)		117(10.55)	206(19.68)		120(10.82)	221(21.11)		
Marital Status, %										
Ever/Married	2131(98.84)	2131(98.84)		1091(98.38)	1091(98.38)		1040(99.33)	1040(99.33)		
Single/Never Married	25(1.16)	25(1.16)		18(1.62)	18(1.62)		7(0.67)	7(0.67)		
Geographical residence, %			<0.001			0.001			<0.001	0.368
Rural	1136(52.69)	965(44.76)		565(50.95)	486(43.82)		571(54.54)	479(45.75)		
Urban	1020(47.31)	1191(55.24)		544(49.05)	623(56.18)		476(45.46)	568(54.25)		
Smoking Habit, %			<0.001			<0.001			<0.001	<0.001
No smoking	1210(56.13)	1157(53.66)		1032(93.06)	1008(90.89)		178(17.00)	149(14.23)		
Currently smoking	784(36.36)	758(35.16)		45(4.06)	64(5.77)		739(70.58)	694(66.28)		
Former smoking	162(7.51)	241(11.18)		32(2.88)	37(3.34)		130(12.42)	204(19.49)		
Using Diabetes Medication, %			<0.001			0.001			0.008	0.412
No	2156(100)	2138(99.17)		1109(100)	1098(99.01)		1047(100)	1040(99.33)		
Yes	0(0)	18(0.83)		0(0)	11(0.99)		0(0)	7(0.67)		
Using Anemia Medication, %			<0.001			0.001			0.009	0.857
No	2147(99.58)	2118(98.24)		1106(99.73)	1090(98.29)		1041(99.43)	1028(98.19)		
Yes	9(0.42)	38(1.76)		3(0.27)	19(1.71)		6(0.57)	19(1.81)		
Using Cholesterol Medication, %			<0.001			0.001			0.001	0.646
No	2155(99.95)	2131(98.84)		1108(99.91)	1095(98.74)		1047(100)	1036(98.95)		
Yes	1(0.05)	25(1.16)		1(0.09)	14(1.26)		0(0)	11(1.05)		
Abdominal obesity ^c^, %			<0.001			<0.001			<0.001	<0.001
No	1383(64.15)	1200(55.66)		488(44.00)	370(33.36)		895(85.48)	830(79.27)		
Yes	773(35.85)	956(44.34)		621(56.00)	739(66.64)		152(14.52)	217(20.73)		
Overweight ^d^, %			0.002			0.014			0.038	<0.001
No	1564(72.54)	1470(68.18)		694(62.58)	637(57.44)		870(83.09)	833(79.56)		
Yes	592(27.46)	686(31.82)		415(37.42)	472(42.56)		177(16.91)	214(20.44)		
Hypertension ^e^, %			0.062			0.082			0.373	<0.001
No	1199(55.61)	1138(52.78)		564(50.86)	523(47.16)		635(60.65)	615(58.74)		
Yes	957(44.39)	1018(47.22)		545(49.14)	586(52.84)		412(39.35)	432(41.26)		
Diabetes, %			<0.001			<0.001			<0.001	0.070
No	2156(100)	2113(98.01)		1109(100)	1081(97.48)		1047(100)	1032(98.57)		
Yes	0(0)	43(1.99)		0(0)	28(2.52)		0(0)	15(1.43)		
Coronary Heart Disease, %			0.000			0.014			0.008	0.704
No	2156(100)	2143(99.40)		1109(100)	1103(99.46)		1047(100)	1040(99.33)		
Yes	0(0)	13(0.60)		0(0)	6(0.54)		0(0)	7(0.67)		
Stroke, %			0.014			0.084			0.084	0.944
No	2156(100)	2150(99.72)		1109(100)	1106(99.73)		1047(100)	1044(99.71)		
Yes	0(0)	6(0.28)		0(0)	3(0.27)		0(0)	3(0.29)		
BMI (kg/m^2^), mean	22.97(4.18)	23.28(4.26)	0.016	23.74(4.50)	24.22(4.63)	0.013	22.15(3.64)	22.29(3.58)	0.380	<0.001
ABSI (m^11/6^ kg^−2/3^), mean	0.0810(0.0066)	0.0830(0.0059)	<0.001	0.0818(0.0072)	0.0843(0.0065)	<0.001	0.0801(0.0059)	0.0817(0.0047)	<0.001	<0.001
WC (cm), mean	80.94(10.78)	83.72(11.83)	<0.001	82.08(11.49)	85.61(12.57)	<0.001	79.72(9.85)	81.73(10.64)	<0.001	<0.001
SBP (mmHg), mean	135.55(21.12)	144.56(25.05)	<0.001	136.88(22.67)	146.37(26.48)	<0.001	134.13(19.25)	142.65(23.30)	<0.001	0.001
DBP (mmHg), mean	81.93(11.70)	83.90(13.38)	<0.001	82.10(12.21)	84.11(13.18)	<0.001	81.75(11.12)	83.68(13.58)	<0.001	0.449

Abbreviation: ABSI, a body shape index; BMI, body mass index; WC, waist circumference; SBP, systolic blood pressure; DBP, diastolic blood pressure; SD, standard deviation. Categorical data are presented as n (%) and continuous data are presented as mean (SD). ^a^ *T*-test was used to compare between year 2007 and 2014 with significance *p*-value < 0.05. ^b^ *T*-test was used to compare between women and men with significance *p*-value < 0.05. ^c^ Abdominal obesity was defined as being >80 cm for women or >90 cm for men. ^d^ Overweight was defined if BMI being ≥25.1 kg/m^2^ and nonoverweight if BMI being <25.1 kg/m^2^. ^e^ Hypertension categorical data (hypertensive vs. nonhypertensive) as an independent variable. Hypertensive defined as systolic blood pressure ≥140 mmHg or diastolic blood pressure ≥90 mmHg.

**Table 2:** Percentage and mean of a body shape index (ABSI) of participants based on the food consumption group in 2007 and 2014.

FCG	2007			2014		
	All	Women	Men	All	Women	Men
**Percentages of participants based on the food consumption group in 2007 and 2014**						
Poor	202(9.37)	115(56.93)	87(43.07)	514(23.84)	286(55.64)	228(44.36)
Borderline	369(17.12)	199(53.93)	170(46.07)	692(32.10)	348(50.29)	344(49.71)
Acceptable	1585(73.52)	795(50.16)	790(49.84)	950(44.06)	475(50.00)	475(50.00)
**Mean ABSI of participants based on the food consumption group in 2007 and 2014**						
Poor	0.0800(0.0071)	0.0813(0.0077)	0.0784(0.0060)	0.0832(0.0062)	0.0846(0.0065)	0.0814(0.0051)
Borderline	0.0809(0.0067)	0.0820(0.0074)	0.0796(0.0568)	0.0830(0.0060)	0.0844(0.0066)	0.0817(0.0495)
Acceptable	0.0811(0.0065)	0.0818(0.0071)	0.0804(0.0059)	0.0830(0.0056)	0.0840(0.0064)	0.0819(0.0044)

Abbreviation: ABSI, a body shape index; FCG, food consumption group. ABSI continuous variable presented as mean (SD) in m^11/6^ kg^−2/3^.

**Table 3:** General estimating equations (GEE) result between food security, ABSI, and hypertension.

Dependent	Independent	β Coef.	CI	*p*-Value
SBP	FCS ^b^	−0.15	(−0.18, −0.12)	<0.001
	FCG ^c^	−3.61	(−4.53, −2.69)	<0.001
	FS ^d^	−5.67	(−7.09, −4.25)	<0.001
	ABSI	32.59	(21.52, 43.66)	<0.001
DBP	FCS ^b^	−0.04	(−0.05, −0.02)	<0.001
	FCG ^c^	−0.85	(−1.35, −0.36)	0.001
	FS ^d^	−1.29	(−2.05, −0.53)	0.001
	ABSI	7.58	(1.66, 13.51)	0.012
HTN ^a^	FCS ^b^	−1.31 × 10^−3^	(−1.99 × 10^−3^, −6.10 × 10^−4^)	<0.001
	FCG ^c^	−3.23 × 10^−2^	(−5.19 × 10^−2^, −1.27 × 10^−2^)	0.001
	FS ^d^	−0.06	(−0.09, −0.03)	<0.001
	ABSI	6.45	(4.11, 8.78)	<0.001
ABSI	FCS ^b^	−1.51 × 10^−5^	(−2.39 × 10^−5^, −6.24 × 10^−6^)	0.001
	FCG ^c^	−2.86 × 10^−4^	(−5.36 × 10^−4^, −3.70 × 10^−5^)	0.024
	FS ^d^	−4.94 × 10^−4^	(−8.78 × 10^−4^, −1.09 × 10^−4^)	0.012

Abbreviations: ABSI, a body shape index; FCS, food consumption score; FCG, food consumption group; FS, food security; HTN, hypertension; DBP, diastolic blood pressure; SBP, systolic blood pressure; CI, confidence interval; β coef., β coefficient. Generalized estimating equation (GEE) test was used an independent variable (2007 & 2014) and a dependent variable (2007 & 2014) with family (Gaussian) link (identity) correlation (independent). ^a^ Hypertension categorical data (hypertensive vs. non-hypertensive) as an independent variable. Hypertensive defined as systolic blood pressure ≥140 mmHg or diastolic blood pressure ≥90 mmHg. ^b^ Food consumption score was used as a continuous data. ^c^ Food consumption group categorized as poor, borderline, and acceptable. ^d^ Food security level categorized as food secure, food insecure.

**Table 4:** Mediation analysis between food security as an independent variable, ABSI as a mediator variable, and hypertension as a dependent variable.

Dependent	Independent	Mediator	Independent → Mediator			Independent → Dependent			Independent → Mediator → Dependent		
			β Coef.	CI	*p*-Value	β Coef.	CI	*p*-Value	β Coef.	CI	*p*-Value
**SBP**	**FCS** ^b^	**ABSI**	−1.51 × 10^−5^	(−2.39 × 10^−5^, −6.24 × 10^−6^)	0.001	−1.53 × 10^−1^	(−0.18, −0.12)	<0.001	−1.48 × 10^−1^	(−0.18, −0.12)	<0.001
	**FCG** ^c^	**ABSI**	−2.86 × 10^−4^	(−5.36 × 10^−4^, −3.70 × 10^−5^)	0.024	−3.61	(−4.53, −2.69)	<0.001	−3.52	(−4.45, −2.60)	<0.001
	**FS** ^d^	**ABSI**	−4.94 × 10^−4^	(−8.78 × 10^−4^, −1.09 × 10^−4^)	0.012	−5.67	(−7.09, −4.25)	<0.001	−5.52	(−6.93, −4.10)	<0.001
**DBP**	**FCS** ^b^	**ABSI**	−1.51 × 10^−5^	(−2.39 × 10^−5^, −6.24 × 10^−6^)	0.001	−0.04	(−0.05, −0.02)	<0.001	−0.03	(−0.05, −0.17)	<0.001
	**FCG** ^c^	**ABSI**	−2.86 × 10^−4^	(−5.36 × 10^−4^, −3.70 × 10^−5^)	0.024	−8.51 × 10^−1^	(−1.35, −0.36)	0.001	−8.30 × 10^−1^	(−1.32, −0.33)	0.001
	**FS** ^d^	**ABSI**	−4.94 × 10^−4^	(−8.78 × 10^−4^, −1.09 × 10^−4^)	0.012	−1.29	(−2.05, −0.53)	0.001	−1.25	(−2.02, −0.49)	0.001
**HTN** ^a^	**FCS** ^b^	**ABSI**	−1.51 × 10^−5^	(−2.39 × 10^−5^, −6.24 × 10^−6^)	0.001	−1.31 × 10^−3^	(−2.00 × 10^−3^, −6.13 × 10^−4^)	<0.001	−1.21 × 10^−3^	(−1.90 × 10^−3^, −5.20 × 10^−4^)	0.001
	**FCG** ^c^	**ABSI**	−2.86 × 10^−4^	(−5.36 × 10^−4^, −3.70 × 10^−5^)	0.024	−3.23 × 10^−2^	(−5.19 × 10^−2^, −1.27 × 10^−2^)	0.001	−3.05 × 10^−2^	(−5.00 × 10^−2^, −1.09 × 10^−2^)	0.002
	**FS** ^d^	**ABSI**	−4.94 × 10^−4^	(−8.78 × 10^−4^, −1.09 × 10^−4^)	0.012	−0.06	(−0.09, −0.03)	<0.001	−0.05	(−0.08, −0.02)	0.001

Abbreviations: ABSI, a body shape index; FCS, food consumption score; FCG, food consumption group; FS, food security; HTN, hypertension; DBP, diastolic blood pressure; SBP, systolic blood pressure; CI, confidence interval; β coef., β coefficient. Mediation analysis test was used an independent variable (2007 & 2014) and a dependent variable (2007 & 2014). ^a^ Hypertension categorical data (hypertensive vs. non-hypertensive) as an independent variable. Hypertensive defined as systolic blood pressure ≥140 mmHg or diastolic blood pressure ≥90 mmHg. ^b^ Food consumption score was used as a continuous data. ^c^ Food consumption group categorized as poor, borderline, and acceptable. ^d^ Food security level categorized as food secure, food insecure.

## Supplementary Materials

The following are available online at http://www.mdpi.com/1660-4601/15/1/175/s1, Table S1: The percentages in food consumption score changes between 2007 and 2014.
