# Supplementary material for: The Association of Food Consumption Scores, Body Shape Index, and Hypertension in a Seven-Year Follow-Up among Indonesian Adults: A Longitudinal Study
Source: Int J Environ Res Public Health. 2018 Jan 22;15(1):175. doi: 10.3390/ijerph15010175 (PMC5800274; doi:10.3390/ijerph15010175)
Supplement: Supplementary file 1 [file ijerph-15-00175-s001.pdf]

International Journal of *Environmental Research and Public Health*

MDPI

Supplementary Table S1. The percentages in food consumption score changes between 2007 and 2014.

FCS	All				Women				Men			
	25th	Median	75th	Mean	25th	Median	75th	Mean	25th	Median	75th	Mean
**2007**	35	49	65	50.76	34	47	64	49.64	36	51	66	51.95
**2014**	22	34	42	33.77	21	33	42	33.29	23	35	42	34.29

FCS changes	% of Participants in Each Category		
	All	Women	Men
Increase	432(20.04)	235(21.19)	197(18.82)
Maintain	39(1.81)	17(1.53)	22(2.10)
Decreased	1685(78.15)	857(77.28)	828(79.08)

Abbreviations: FCS, food consumption score. The FCS change is presenting in n(%).
